# Role of peri-deployment right ventricular paced-ECG in left bundle area pacing

**DOI:** 10.3389/fcvm.2025.1555683

**Published:** 2025-09-10

**Authors:** Wen-De Tang, Chiung-Ray Lu, Mei-Yao Wu, Ching-Fen Chang, Wei-Hsin Chung, Yin-Huei Chen, Hung-Pin Wu, Hei-Tung Yip, Kuan-Cheng Chang, Yen-Nien Lin

**Affiliations:** ^1^ Division of Cardiovascular Medicine, Department of Internal Medicine, China Medical University and Hospital, Taichung, Taiwan; ^2^ Department of Chinese Medicine, China Medical University Hospital, Taichung, Taiwan; ^3^ Division of Endocrinology and Metabolism, Department of Internal Medicine, China Medical University Hospital, Taichung, Taiwan; ^4^ Management Office for Health Data, China Medical University Hospital, Taichung, Taiwan; ^5^ College of Medicine, China Medical University, Taichung, Taiwan; ^6^ School of Medicine, China Medical University, Taichung, Taiwan

**Keywords:** conduction system pacing, ECG, pacemaker, left bundle pacing, deep septal pacing

## Abstract

**Background:**

Left bundle area pacing (LBBAP) has emerged as a promising physiological pacing modality. The current technique for LBBAP lead implantation predominantly relies on the anatomy of the His bundle and right ventricular septum. While practical, this anatomical approach can lead to variations in lead polarity and QRS durations due to the relatively extensive target zone.

**Objectives:**

This study aims to investigate whether peri-deployment paced ECGs can effectively guide LBBAP and enhance left ventricular activation.

**Methods:**

We conducted a retrospective analysis of 41 patients (54 attempts) who underwent LBBAP between 1 September 2020 and 30 June 2021. We collected and analyzed demographic data, baseline ECGs, intraprocedural ECGs, and ventricular local electrograms. QRS patterns were categorized into five common types, R, Rs, rs, rS, and S, and were labeled from 1 to 5 for polarity analysis. In addition, we explored markers associated with achieving shorter QRS durations (<120 ms).

**Results:**

LBBAP was successfully achieved in 81.5% of the attempts. During the procedure, the paced QRS duration (QRSd) significantly decreased from 144.5 ± 22.6 ms–128.8 ± 22.9 ms (*p* < 0.001). Comparing lead polarity scores before and after deployment revealed a significant increase in leads I and aVL (lead I, 1.3 ± 0.9 vs. 1.6 ± 1.0, *p* = 0.002; lead aVL, 1.6 ± 1.0 vs. 2.1 ± 1.3, *p* = 0.002), while leads III and aVF showed a decrease (lead III, 3.9 ± 1.2 vs. 3.4 ± 1.5, *p* = 0.001; lead aVF, 3.1 ± 1.2 vs. 2.9 ± 1.3, *p* = 0.026). The polarity of leads II and aVR remained unchanged. In the subgroup with post-deployment QRSd shorter than 120 ms, although the Qr pattern in lead V1 was only numerically higher (95.2% vs. 81.8%, *p* = 0.310), the lead polarity scores were significantly higher in leads I and aVL and lower in leads III and aVF (*p* < 0.001). This group also had a significantly shorter left ventricular activation time (LVAT) (68.7 ± 13.0 ms vs. 98.4 ± 14.0 ms, *p* < 0.001). Univariate analysis revealed that a shorter pre-deployment paced QRSd and LVAT were associated with a narrower post-deployment QRSd. In addition, non-electrical factors such as female gender and left ventricular dilation were associated with higher post-deployment QRSd.

**Conclusions:**

Peri-deployment ECG assessment is a practical adjunct to anatomy-based LBBAP, providing real-time markers for optimal lead positioning. Specifically, an unaltered lead II axis and expected changes in the lead I/aVL and lead III/aVF axes can help guide the selection of the left bundle branch. Lower pre-deployment paced QRSd and LVAT, as well as a more rightward inferior axis after deployment, are associated with a shorter post-deployment QRSd.

## Introduction

Conduction system pacing (CSP) is a pioneering technique designed to directly activate the ventricles by capturing the His bundle (His bundle pacing, HBP) or the left bundle branch (left bundle branch area pacing, LBBAP). This approach was developed to prevent the adverse effects of pacing-induced desynchrony associated with traditional right ventricular pacing (1–3). CSP has demonstrated significant benefits in improving left ventricular (LV) synchrony and ejection fraction in heart failure patients with left bundle branch block (LBBB), establishing its novel therapeutic role in cardiac resynchronization (1, 4–6). Among CSP techniques, LBBAP has emerged as the preferred method due to its several advantages over HBP. LBBAP consistently offers lower pacing thresholds, higher sensing amplitudes, more stable lead positioning, and comparable physiological pacing effects. These attributes have led to its widespread acceptance as the most effective CSP technique (7, 8).

The current technique for LBBAP lead implantation is generally guided by the anatomy of the His bundle and right ventricular septum, with some modifications (9–14). Unlike the His bundle area (HBA), the left bundle branches (LBBs) penetrate the LV septum, forming a wider target area for pacing (15, 16). The target zone for LBBAP, rather than being strictly 1.5–2.0 cm from the His bundle in the apical direction as described by Huang et al., is considered more liberally and includes a wide area on the midseptum (9, 10). The LBBAP lead deployment site is typically determined using markers such as the His bundle (10, 11), tricuspid ring (12, 13), paced QRS morphology (polarity discordance of leads II and III, and V1 nadir notch), and endocardial electrograms (EGMs) (7). Consistent with this approach, Jiang et al. described a nine-partition method for guiding LBBAP in a specific zone, contributing to 10%–20% of the right ventricular septum (14, 17). Although the anatomical approach for LBBAP is feasible, the relatively large target zone can lead to variable lead polarities and QRS durations, resulting in multiple lead implantation attempts (18, 19).

Electrocardiography (ECG) plays an indispensable role in LBBAP. The dynamic changes in paced QRS morphology, especially in lead V1, during implantation reflect the lead depth in the interventricular septum (20). Certain ECG markers, such as R-wave peak time in V6 (10, 21), the V6–V1 interpeak interval (22), and the isoelectric interval (23), are useful for diagnosing conduction system capture (16). Recent studies have classified capture locations within the LV conduction system (proximal LBB or left bundle fascicles) using ECG to investigate clinical outcomes (18, 24, 25). Notably, a right ventricular septal paced ECG is routinely performed before lead deployment. To address the electrical properties of LBBAP more effectively, we investigated ECGs before and after lead deployment. We hypothesized that pre-deployment paced ECGs are beneficial to the LBBAP procedure and complementary to purely anatomy-guided lead deployment. We propose that peri-deployment paced ECGs can guide LBBAP and achieve efficient LV activation.

## Methods

### Study design and patient population

**Figure 1:**
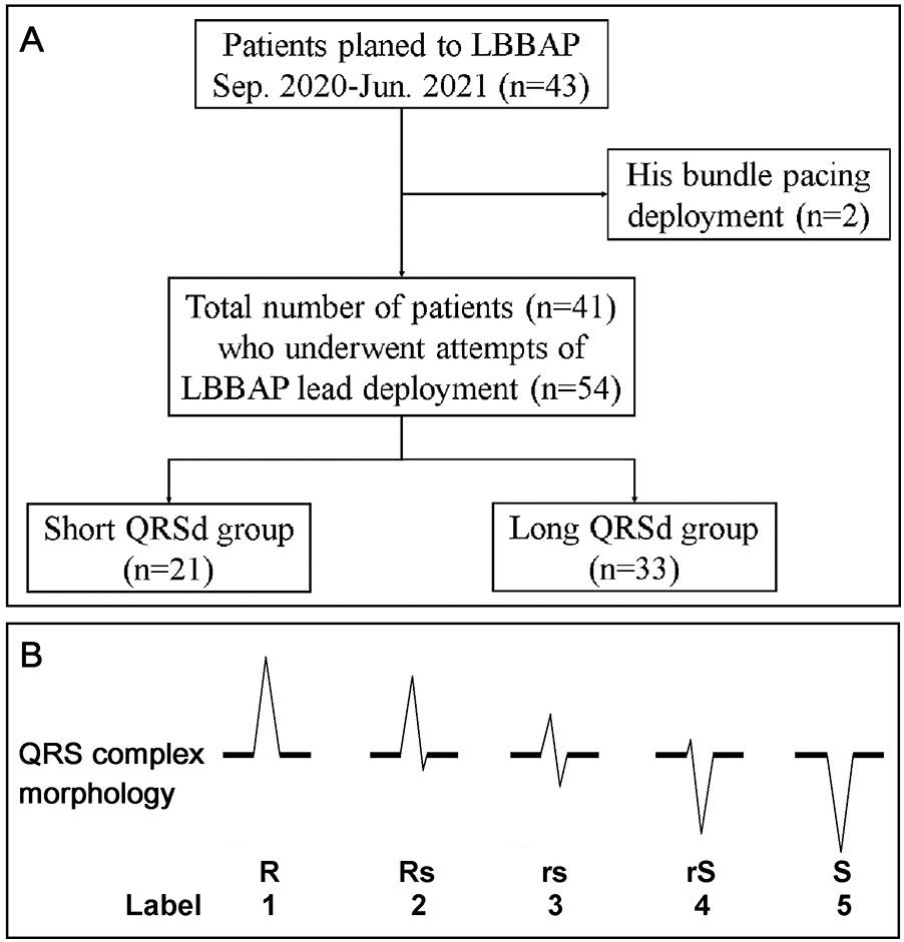
**(A)** Patient flow diagram. Flowchart showing the selection of the final cohort of 41 patients (54 attempts) who underwent LBBAP. **(B)** The morphology of the QRS complex on the surface ECG was categorized into five common subtypes: R, Rs, rs, rS, and S. These subtypes were semiquantitatively labeled from 1 to 5.

We conducted a retrospective analysis of 41 consecutive patients with sinus nodal dysfunction or atrioventricular block who underwent LBBAP at China Medical University Hospital between 1 September 2020 and 30 June 2021. All procedures were performed by four board-certified electrophysiologists with extensive experience in device implantation and physiological pacing, following a standardized implantation protocol to minimize inter-operator variability. The demographic data, baseline ECG, LBBAP ECG, intraprocedural ECG, and ventricular local EGM were collected and analyzed. Shorter QRS duration has long served as a predictor for favorable cardiovascular outcomes in heart failure patients receiving cardiac resynchronization therapy. Although QRS duration is going to be shortened with successful LBBAP, it may still vary from place to place. We categorized the LBBAP QRS duration (QRSd) into two groups: longer QRSd (≥120 ms) and shorter QRSd (<120 ms). We then explored the markers that contributed to achieving a shorter QRSd (Figure 1A). The study protocol was reviewed and approved by the Research Ethics Committee of China Medical University Hospital (IRB: CMUH110-REC2-114). The committee waived the requirement for written informed consent due to the retrospective nature of the database research design.

### Left bundle branch area pacing device implantation

Patients underwent the LBBAP procedure under conscious sedation using local anesthesia. The procedure was typically performed via a transeptal approach as previously described (7, 10, 16), utilizing the pacing lead (Select Secure Model 3830, 69 cm, Medtronic Inc., Minneapolis, MN, USA) and a fixed curve sheath (C315HIS, Medtronic Inc., Minneapolis, MN, USA). A 12-lead surface ECG and the EGM from the pacing lead were displayed and recorded on a multichannel recorder (GE ComboLab Electrophysiology and Hemodynamic Recording System). The LBBAP lead deployment site was determined using the His bundle and tricuspid ring as landmarks or the nine-partition method. In addition, paced QRS morphology was assessed for the presence of a V1 nadir notch. The lead was advanced into the interventricular septum until the criteria for LBBAP were met. However, operators were allowed to adjust the lead depth based on the patient's medical condition during the procedure.

### Definition of successful LBBAP and algorithm for confirming conduction system capture with LBBAP

Successful LBBAP pacing was defined by the presence of a right bundle branch delay pattern in ECG lead V1 during lead tip pacing. Further differentiation of left bundle branch (LBB) capture from left ventricular septal pacing (LVSP) within the LBBAP area was based on specific criteria: (1) QRS transition from LVSP to selective LBBP during threshold test and (2) a V6 R-wave peak time or peak LV activation time <80 ms. Likely LBBP was defined as a V6 R-wave peak time or peak LV activation time <85 ms for patients with native narrow QRS and isolated right bundle branch block (RBBB) and <100 ms for those with LBBB and intraventricular conduction delay (IVCD). Pacing that did not meet these criteria for LBB capture was classified as LVSP (10, 16). Echocardiography was utilized to confirm that the lead tip was located just under the left-side sub-endocardium of the interventricular septum. Unsuccessful LBBAP, also referred to as deep septal pacing, was defined as the failure to meet all of the above criteria (16).

### Periprocedure ECG and semi-quantification of the paced QRS complex

Periprocedure ECGs were recorded before, during, and after LBBAP lead deployment. ECGs were routinely monitored before and during the lead deployment during ventricular lead unipolar pacing at 5 V. After the lead fixation, a decremental output test was performed for evaluation of the threshold and capturing selectivity. To maintain consistency in ECG analysis across different patients and attempts, ECGs obtained from unipolar pacing at 5 V were recorded for analysis. The post-procedure ECGs were recorded using unipolar pacing at the lowest output that captured the myocardium or the conduction system. We semi-quantified the QRS morphology to facilitate polarity analysis and potential future application during the procedure. The QRS patterns were categorized into five common subtypes, R, Rs, rs, rS, and S, and were labeled from 1 to 5 (Figure 1B). In addition, the precordial transition zone was identified. Peak left ventricular activation time (pLVAT) was estimated by measuring the time from the pacing spike to the R-wave peak of a QRS complex in precordial leads V5 and V6, according to previous reports (26, 27). The difference between pre-deployment LVAT and post-deployment LVAT was defined as ΔpLVAT (ΔpLVAT = pLVAT_post-deploy_ − pLVAT_pre-deploy_).

### Statistical analysis

Continuous variables were reported as mean ± standard deviation (SD), while categorical variables were expressed as numbers with percentages. The differences between the two groups were assessed using the Wilcoxon test for continuous variables and Fisher’s exact test for categorical variables. Differences between pre- and post-deployment data were analyzed using the paired sample *t*-test for continuous variables and the paired samples Wilcoxon test for categorical variables. A logistic regression model was applied to estimate the odds ratio. A two-tailed probability value of <0.05 was considered statistically significant. All statistical analyses were performed using R software (version 4.0).

## Results

### Patient characteristics

All eligible patients during the study period were enrolled, resulting in a total of 41 consecutive cases. The mean age was 69.4 ± 11.5 years, and 51.2% of the participants were male (Table 1). We performed 54 attempts of LBBAP lead deployment on these patients. The most common comorbidities among them were hypertension (78.0%), chronic kidney disease (stage III–V, 51.2%), and diabetes (48.8%). The pacing indications for these patients were varied, including atrioventricular block (41.5%), atrial fibrillation with slow ventricular response (7.3%), sick sinus syndrome (12.2%), and cardiac resynchronization therapy (39.0%). Baseline left ventricular ejection fraction (LVEF), as measured by echocardiography, was 56.0 ± 13.6%. The left ventricular end-diastolic diameter (LVEDD) and interventricular septum measured 52.7 ± 8.6 mm and 9.1 ± 6.3 mm, respectively. The initial QRSd was 109.2 ± 27.0 ms. In addition, the percentages of patients with right bundle branch block, left bundle branch block, and IVCD were 14.6%, 14.6%, and 4.9%, respectively.

**Table 1:** Patient characteristics.

	All patients (*n* = 41)
Age, years	69.4 ± 11.5
Female sex	20 (48.8)
Comorbidities	
Coronary artery disease	10 (24.4)
Hypertension	32 (78.0)
Diabetes mellitus	20 (48.8)
Chronic kidney disease	21 (51.2)
Atrial arrhythmia	17 (41.5)
Indications	
Sick sinus syndrome	5 (12.1)
Atrioventricular block	17 (41.5)
AF with slow ventricular response	3 (7.3)
Cardiac resynchronization therapy	16 (39.0)
Echocardiography	
LVEF, %	56.0 ± 13.6
LVEDD, mm	52.7 ± 8.6
IVS, mm	9.1 ± 6.3
Baseline ECG	
QRSd, ms	109.2 ± 27.0
RBBB, *n* (%)	6 (14.6)
LBBB, *n* (%)	6 (14.6)
IVCD, *n* (%)	2 (4.9)

Values are presented as mean ± SD or *n* (%). AF, atrial fibrillation; LVEF, left ventricular ejection fraction; LVEDD, left ventricular end-diastolic diameter; IVS, interventricular septum; QRSd, QRS duration; RBBB, right bundle branch block; LBBB, left bundle branch block; IVCD, intraventricular conduction delay.

### Changes of electrograms and paced ECGs before and after LBBAP lead deployment

Before lead deployment, paced ECGs recorded at the right ventricular septum showed a QRSd of 144.5 ± 22.6 ms, characterized by a V1 nadir notch (W pattern). The score of lead polarity was lead II 2.0 ± 1.0, lead III 3.9 ± 1.2, and aVF 3.1 ± 1.3, compatible with inferior polarity discordance. The QRS transition zone was observed after V3 (3.4 ± 1.3), and the pLVAT was measured at 97.4 ± 22.9 ms.

After leads were advanced to the interventricular septum, LBBAP was successfully achieved in 81.5% LBBAP attempts. In the short QRS group, the success rate was 95.2% with 70% in LBBP and 23.8% in likely LBBP. While in the long QRS group, the success rate was lower (85.2%). Only 14.7% attempts were LBBP, 20.5% were likely LBBP, and 50% were LVSP. Following lead deployment, the QRSd significantly decreased to 128.8 ± 22.9 ms (*p* < 0.001, Table 2), and the pLVAT was also significantly reduced to 87.1 ± 20.4 ms (*p* = 0.001). The post-deployment precordial transition occurred after V2 (2.9 ± 1.3). Despite the post-deployment ECG remaining inferior discordant, notable changes in the polarity of certain leads were observed. Specifically, the polarity scores of leads I and aVL increased (lead I, 1.3 ± 0.9 vs. 1.6 ± 1.0, *p* = 0.002; lead aVL, 1.6 ± 1.0 vs. 2.1 ± 1.3, *p* = 0.002), while the scores for leads III and aVF decreased significantly (lead III, 3.9 ± 1.2 vs. 3.4 ± 1.5, *p* = 0.001; lead aVF, 3.1 ± 1.2 vs. 2.9 ± 1.3, *p* = 0.026). Notably, the polarity of leads II and aVR remained unchanged after lead deployment.

**Table 2:** Electrocardiographic characteristics between before and after LBBAP lead deployment.

	Pre-deployment	Post-deployment	*p*
	(*n* = 54)	(*n* = 54)	
QRS duration, ms	144.5 ± 22.6	128.8 ± 22.9	<0.001
I	1.3 ± 0.9	1.6 ± 1.0	0.002
II	2.0 ± 1.0	1.8 ± 1.0	0.435
III	3.9 ± 1.2	3.4 ± 1.5	0.001
aVR	4.4 ± 1.0	4.6 ± 0.8	0.450
aVL	1.6 ± 1.0	2.1 ± 1.3	0.002
aVF	3.1 ± 1.2	2.6 ± 1.4	0.026
Precordial transition	3.4 ± 1.3	2.9 ± 1.3	0.016
V1 morphology			<0.001
W	54 (100)	7 (13.0)	
Qr	0 (0)	47 (87.0)	
pLVAT, ms	97.4 ± 22.9	87.1 ± 20.4	0.001

Values are presented as mean ± SD or *n* (%). pLVAT, peak left ventricular activation time.

### Differences in pre-deployment paced ECGs between post-deployment QRS duration greater or less than 120 ms

**Figure 2:**
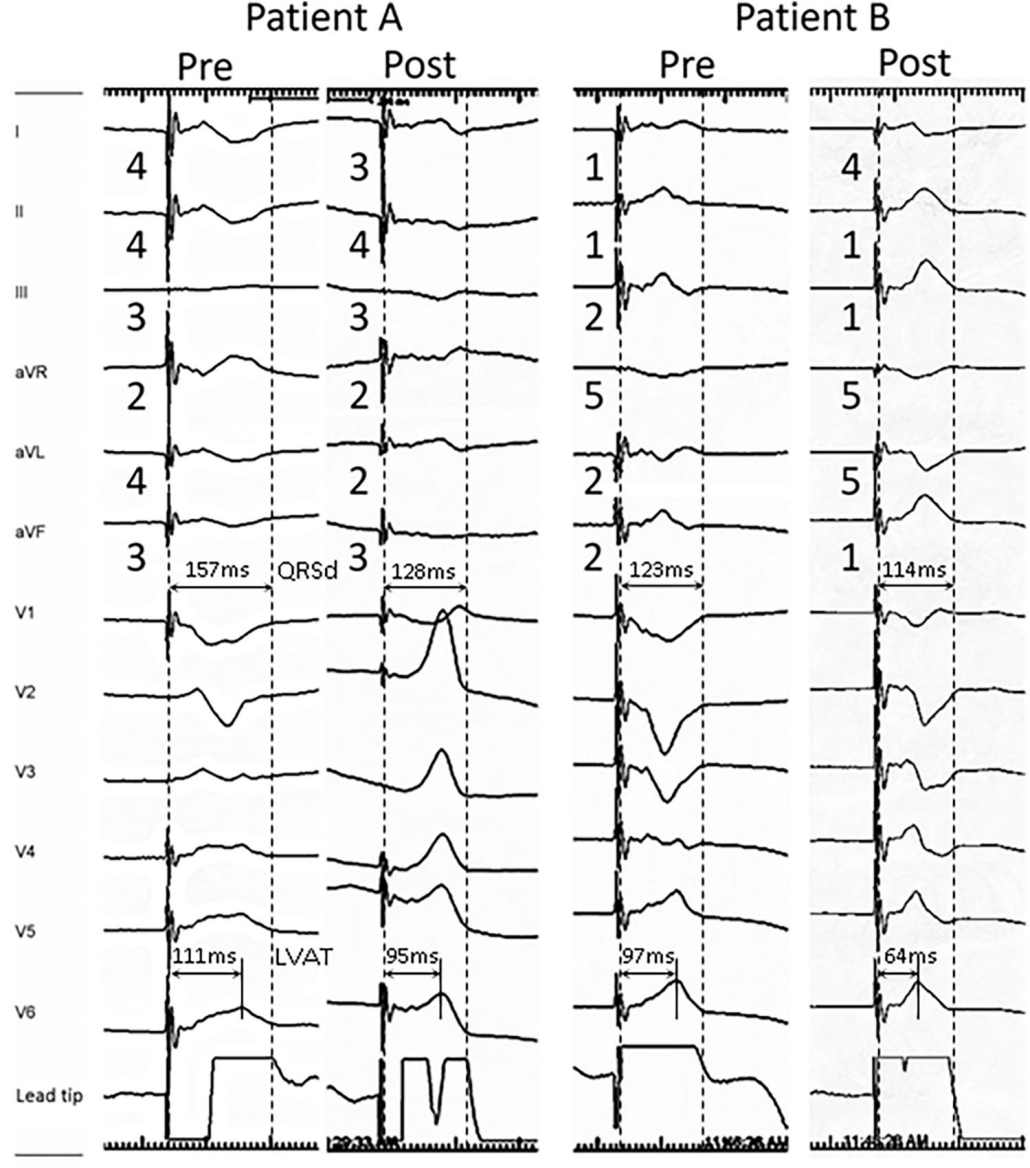
Representative 12-lead ECG morphology change during LBBAP lead deployment in patients with post-deployment QRSd ≧ 120 ms (patient **A**) and QRSd < 120 ms (patient **B**). The limb lead polarity of leads II and aVR remained unchanged after lead deployment in both patients. Post-deployment polarity decreased in leads I and aVL, and increased in leads III and aVF. Both patients presented Qr pattern in V1, while patient B had a rightward inferior axis in post-deployment ECG and lower pre-deployment LVAT (111 vs. 97 ms) and QRSd (157 vs. 123 ms), which were associated with lower post-deployment QRSd (128 vs. 114 ms).

The QRS complex represents the process of ventricular depolarization, with an increased QRSd suggesting dyssynchronous contraction and relaxation of the left and right ventricles (1, 28). We stratified LBBAP attempts by post-deployment QRSd 120 ms. Of the total 54 attempts, 21 of them had a QRSd of <120 ms (short QRSd group), with a mean duration of 104.7 ± 8.4 ms, while the remaining attempts had a QRSd of 120 ms or more (long QRSd group), with a mean duration of 142.1 ± 16.5 ms (Table 3, Figure 2). In comparison to the long QRSd group, the pre-deployment paced QRS in the short QRSd group was significantly narrower (132.2 ± 13.8 ms vs. 151.4 ± 23.7 ms, *p* < 0.001). In addition, the pre-deployment pLVAT was significantly shorter in the short QRSd group compared with the long QRSd group (83.0 ± 17.5 ms vs. 106.6 ± 21.3 ms, *p* < 0.001). The polarity scores of the limb leads also differed between the two groups, particularly in leads aVR and aVL. The short QRSd group exhibited a lower polarity score in lead aVR (4.2 ± 1.2 vs. 4.6 ± 0.9, *p* < 0.001) and a higher polarity score in lead aVL (1.9 ± 1.1 vs. 1.5 ± 0.9, *p* < 0.001), suggesting a rightward axis. Furthermore, the precordial transition occurred later in the short QRSd group compared with the long QRSd group (3.8 ± 1.1 vs. 3.2 ± 1.3, *p* < 0.001).

**Table 3:** Comparison of pre-deployment paced ECGs between the short and long QRSd groups.

Pre-deployment paced ECGs	Short QRSd group	Long QRSd group	*p*
	(*n* = 21)	(*n* = 33)	
QRS duration, ms	132.2 ± 13.8	151.4 ± 23.7	<0.001
I	1.4 ± 0.9	1.3 ± 0.9	<0.001
II	1.8 ± 1.2	2.1 ± 1.0	<0.001
III	3.6 ± 1.2	4.0 ± 1.2	<0.001
aVR	4.2 ± 1.2	4.6 ± 0.9	<0.001
aVL	1.9 ± 1.1	1.5 ± 0.9	<0.001
aVF	3.0 ± 1.2	3.2 ± 1.3	<0.001
Precordial transition	3.8 ± 1.1	3.1 ± 1.4	<0.001
V1 morphology			-
W	21 (100)	33 (100)	
Qr	0 (0)	0 (0)	
pLVAT, ms	83.0 ± 17.5	106.6 ± 21.3	<0.001

Values are presented as mean ± SD or *n* (%). Short QRSd group = post-deployment QRS duration < 120 ms; Long QRSd group = post-deployment QRS duration ≥ 120 ms; pLVAT, peak left ventricular activation time.

### Differences in post-deployment paced ECGs between post-deployment QRS duration greater or less than 120 ms

Lead deployment is a major factor affecting post-deployment ECGs. After advancing the lead, post-deployment QRSd became 104.7 ± 8.4 ms and 142.1 ± 16.5 ms in short and long QRSd groups, respectively. The pLVAT were 68.7 ± 13.0 ms in the short QRSd group while 98.4 ± 14.0 ms in the long QRSd group (*p* < 0.001) (Table 4, Figure 2). The change of pLVAT was significantly higher in the short QRSd group (−14.3 ± 18.4 vs. −7.8 ± 21.6, *p* < 0.001). In addition, the ratio of right bundle branch delay pattern (Qr) in ECG lead V1 was numerically higher in the short QRSd group, although not statistically significant (95.2% vs. 81.8%, *p* = 0.310). Compared to the long QRSd group, the polarity score of limb leads generally showed a significant rightward inferior axis in the short QRSd group. The precordial transition in the short QRSd group occurred later than in the long QRSd group (3.2 ± 1.3 vs. 2.7 ± 1.3, *p* < 0.001). Interestingly, the short QRSd group exhibited a larger change in polarity scores before and after lead deployment, while the long QRSd group showed only minimal changes.

**Table 4:** Comparison of post-deployment paced ECGs between the short and long QRSd groups.

Post-deployment paced ECGs	Short QRSd group	Long QRSd group	*p*
	(*n* = 21)	(*n* = 33)	
QRS duration, ms	104.7 ± 8.4	143.4 ± 17.2	<0.001
I	2.1 ± 1.1	1.4 ± 0.8	<0.001
II	1.6 ± 1.0	2.0 ± 1.1	<0.001
III	2.3 ± 1.4	3.9 ± 1.3	<0.001
aVR	4.5 ± 0.7	4.6 ± 0.9	<0.001
aVL	2.9 ± 1.3	1.6 ± 1.0	<0.001
aVF	2.1 ± 1.4	3.1 ± 1.3	<0.001
Precordial transition	3.2 ± 1.3	2.7 ± 1.3	<0.001
V1 morphology			0.310
W	1 (4.8)	6 (18.2)	
Qr	20 (95.2)	27 (81.8)	
pLVAT, ms	68.7 ± 13.0	98.9 ± 14.8	<0.001
Δ pLVAT, ms	−14.3 ± 18.4	−7.8 ± 21.6	<0.001

Values are presented as mean ± SD or *n* (%). Short QRSd group = post-deployment QRS duration < 120 ms; Long QRSd group = post-deployment QRS duration ≥ 120 ms; pLVAT, peak left ventricular activation time; Δ pLVAT = pLVAT_post-deploy_ − pLVAT_pre-deploy_.

### Predictors associated with post-deployment QRS duration

Patients with narrow QRS durations tend to exhibit better ventricular synchronization and consistently achieve more favorable outcomes compared with those with wider QRS durations. It is important to determine the predictors associated with post-deployment QRS duration. By univariate analysis, we found that female gender (OR, 4.06; 95% CI, 1.42–14.9; *p* = 0.011), smaller LV chamber size (OR, 0.87; 95% CI, 0.74–0.92; *p* = 0.004), shorter pre-deployment paced QRS duration (OR, 0.95; 95% CI, 0.91–0.98; *p* = 0.003), and shorter pLVAT (OR, 0.94; 95% CI, 0.89–0.97; *p* = 0.001) were associated with a narrower post-deployment QRSd (Table 5). Furthermore, age and precordial transition showed a trend toward association with post-deployment QRS duration, although they did not reach statistical significance. On the other hand, post-deployment QRS duration was not significantly associated with left ventricular ejection fraction (LVEF), LV septal wall thickness, baseline QRS duration, the presence of bundle branch block, or limb lead polarity in patients receiving LBBAP.

**Table 5:** Multivariate logistic regression assessing the impact of variables between the short and long QRSd groups.

Variables	Short QRSd group	Long QRSd group	Odd ratio	*p*
Age, years	74.7 ± 10.0	68.8 ± 12.6	1.05 (1.00,1.11)	0.059
Sex				
Female	14 (58.3)	10 (41.7)	4.6 (1.42, 14.9)	0.011
Male	7 (23.3)	23 (76.7)	1.00	(reference)
Comorbidities				
Coronary artery disease	4 (25)	12 (75)	0.41 (0.11, 1.51)	0.181
Hypertension	16 (36.4)	28 (63.6)	0.57 (0.14, 2.28)	0.428
Diabetes mellitus	11 (40.7)	16 (59.3)	1.17 (0.39, 3.49)	0.780
Chronic kidney disease	11 (37.9)	18 (62.1)	0.92 (0.31, 2.75)	0.876
Atrial arrhythmia	12 (54.5)	10 (45.5)	3.07 (0.98, 9.58)	0.054
Pacing indication				
Sick sinus syndrome	7 (41.2)	10 (58.8)	1.00	(reference)
Atrioventricular block	7 (29.2)	17 (70.8)	0.60 (0.15, 2.28)	0.430
AF with slow ventricular response	6 (66.7)	3 (33.3)	2.69 (0.50, 17.6)	0.216
Cardiac resynchronization therapy	1 (25)	3 (75)	0.53 (0.02, 5.66)	0.549
Echocardiography				
LVEF, %	58.0 ± 10.2	54.0 ± 14.2	1.01 (0.98, 1.08)	0.604
LVEDD, mm	48.4 ± 6.0	56.3 ± 7.7	0.87 (0.74, 0.92)	0.004
IVS, mm	8.3 ± 1.7	9.3 ± 6.9	0.96 (0.74, 1.07)	0.480
Baseline ECG				
QRSd, ms	99.2 ± 25.3	111.9 ± 27.2	0.98 (0.96, 1.00)	0.069
RBBB	3 (42.9)	4 (57.1)	0.83 (0.17, 4.13)	0.818
LBBB	3 (42.9)	4 (57.1)	0.83 (0.17, 4.13)	0.818
IVCD	0 (0.0)	0 (0.0)	NA	
Pre-deployment paced ECGs				
QRS duration, ms	132.2 ± 13.8	152.4 ± 23.7	0.95 (0.91, 0.98)	0.003
I	1.4 ± 0.9	1.3 ± 0.9	1.30 (0.64, 2.41)	0.405
II	1.8 ± 1.2	2.1 ± 1.0	0.90 (0.43, 1.34)	0.690
III	3.6 ± 1.2	4.0 ± 1.2	0.86 (0.45, 1.18)	0.505
aVR	4.2 ± 1.2	4.6 ± 0.9	0.68 (0.40, 1.24)	0.167
aVL	1.9 ± 1.1	1.5 ± 0.9	1.52 (0.92, 2.97)	0.133
aVF	3.0 ± 1.2	3.2 ± 1.3	1.06 (0.92, 2.97)	0.766
Precordial transition	3.8 ± 1.1	3.1 ± 1.4	1.55 (0.97, 2.59)	0.081
pLVAT, ms	83.0 ± 17.5	106.6 ± 21.3	0.94 (0.89, 0.97)	0.001

Values are presented as mean ± SD or *n* (%). Short QRSd group = post-deployment QRS duration < 120 ms; Long QRSd group = post-deployment QRS duration ≥ 120 ms; AF, atrial fibrillation; LVEF, left ventricular ejection fraction; LVEDD, left ventricular end-diastolic diameter; IVS, interventricular septum; QRSd, QRS duration; RBBB, right bundle branch block; LBBB, left bundle branch block; IVCD, intraventricular conduction delay; pLVAT, peak left ventricular activation time.

## Discussion

**Figure 3:**
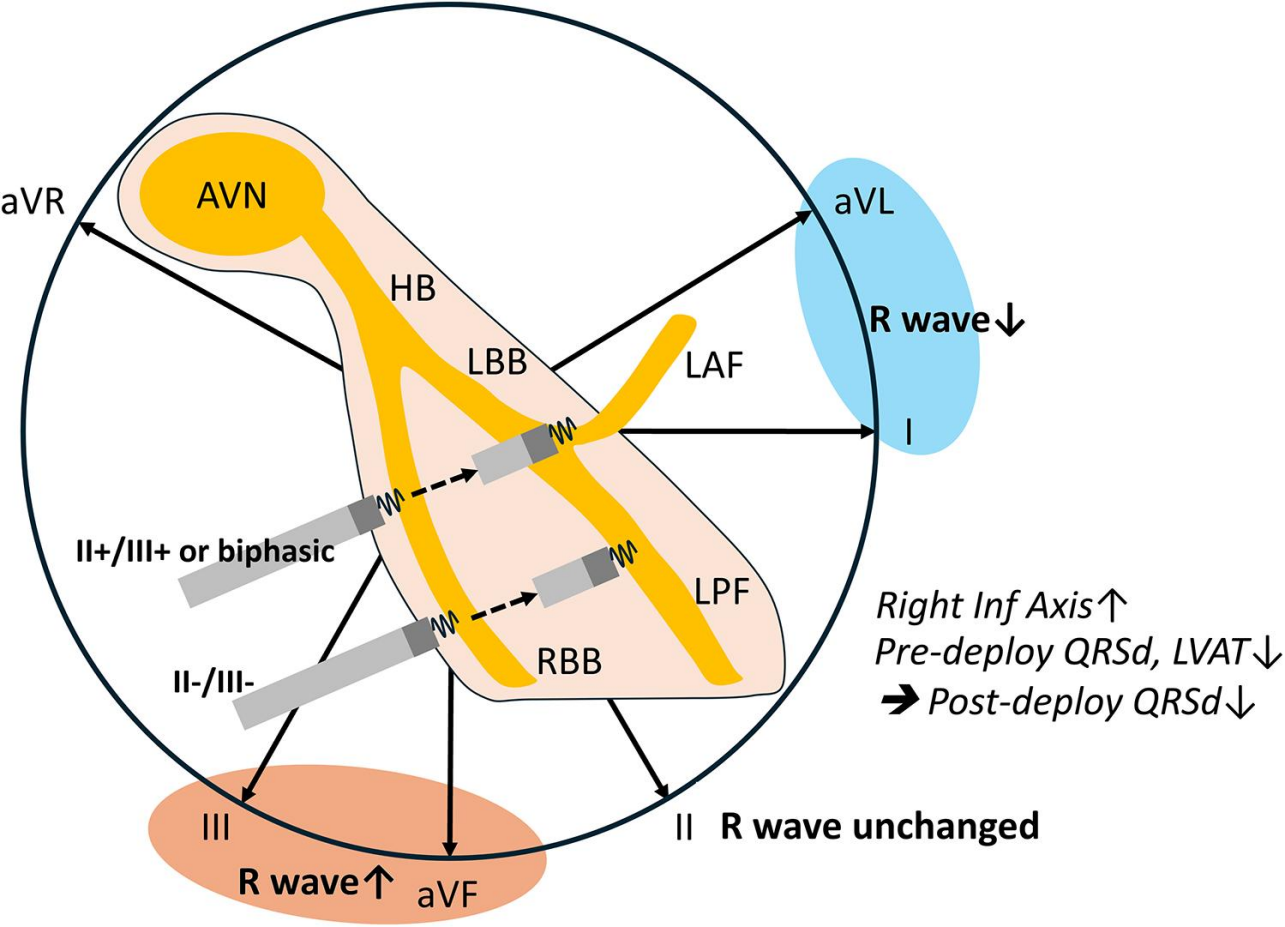
Peri-deployment ECG is helpful for LBBAP. Unaltered lead II axis and anticipated lead I/aVL and lead III/aVF axes change could guide the left bundle selection. This aligns with the anatomical route of the lead advancement. Lower pre-deployment paced QRSd and LVAT and a post-deployment rightward inferior axis are associated with shorter post-deployment QRSd.

The major findings of the current study revealed that the limb lead polarity of leads II and aVR remained unchanged after lead deployment. Anticipated post-deployment polarity decrease in leads I and aVL and increase in leads III and aVF can be useful for optimizing LBB selection (Figure 3). More than the Qr pattern in V1, a rightward inferior axis in post-deployment ECG is associated narrower QRS. Moreover, lower pre-deployment LVAT and QRSd were also predictors of shorter post-deployment QRSd. Certain non-electrical factors were identified as being associated with post-deployment QRS duration. Specifically, male gender and larger left ventricular (LV) chamber size were linked to longer post-deployment QRS durations.

### Pre-deployment paced ECG lead II and III R waves guide the left bundle system selection

The final QRS polarity depends on the specific segment where the lead is deployed. When the lead captures the mid-segment of the LBBAP or the septal fascicle, the ECG typically shows a discordant pattern in leads II and III. If the lead is positioned in the left posterior fascicle, the QRS complexes in leads II, III, and aVF are predominantly negative. In contrast, capture of the left anterior fascicle results in predominantly negative QRS complexes in leads I and aVL. Our study demonstrated that ECG signals, in conjunction with right ventricular (RV) septal anatomy, are valuable for guiding left bundle branch area pacing (LBBAP). Consistent with the direction of ventricular lead advancement in the septum, our data revealed that the R-wave amplitude in leads I and aVL decreased, while the R-wave amplitude in leads III and aVF increased post-deployment (Figure 3). This aligns with the anatomical route of the lead advancement and suggests a higher proportion of left anterior fascicular capture. Notably, the R waves in leads II and aVR remained unchanged, likely because these axes are almost perpendicular to the lead's advancement path. Pre-deployment negative R wave in leads II and III may direct the lead toward the left posterior fascicle (LPF). Conversely, a positive R wave in lead II accompanied by positive or biphasic lead III may guide the lead toward the left bundle branch trunk (LBT) or left anterior fascicle (LAF). Current guides to LBBAP suggest that the ECG signals, inferior lead, and aVR/aVL discordance indicate an ideal location of lead deployment (10, 29). Our study first provides the supported evidence. Recently, research has divided the LBBA of ventricular capture into LBT, LPF, and LAF based on the paced QRS complex (18). The paced QRS complexes in these three groups were relatively narrow, and the LVAT was short. In addition, the pacing thresholds remained stable across these groups (18, 30). Although current evidence indicates that pacing at different sites within the LBB system results in similar intraventricular and interventricular electrical synchrony, the long-term outcomes of pacing at specific areas within the LBB system remain uncertain. Pre-deployment paced ECG, especially leads II and III, remains important and provides additional information that can guide LBBAP.

### Pre-deployment LVAT and lead advancement were associated with post-deployment QRSd

The duration of the QRS complex is a crucial indicator of ventricular activation efficiency. A narrower QRS complex and synchronized ventricular activation are generally associated with better cardiovascular outcomes, particularly in patients with heart failure and LBBB (31, 32). In our study, shorter LVAT paced from the RV septum before lead deployment was associated with shorter post-deployment QRS durations. This suggests that a shorter RV septal paced LVAT might indicate partial capture of the LV from the RV pacing. This phenomenon could be due to a thinner interventricular septum at the pacing site or partial septal advancement of the lead and sheath. Regardless of the reasons, these findings emphasize the importance of targeting a shorter LVAT before advancing the lead. The depth of lead advancement also plays a significant role in determining the QRS duration. Our study showed that a RBBB pattern in V1, rightward inferior ventricular axis, and larger change of limb lead polarity were more common in the short QRS duration group. These ECG findings provide electrical evidence that the closer the lead tip is to the LV sub-endocardium, the shorter the QRS duration after lead deployment. Together, it is essential to advance the lead until the presence of the lead V1 R' pattern and limb lead axis right inferior alternation.

### Non-electrical factors associated with post-deployment QRSd

Careful pre-procedural assessment is essential for successful LBBAP (18). Our study identified several non-electrical factors, including male gender and larger LV chamber size, that were associated with longer post-deployment QRSd and are thought to affect the lead advancement in the septum. Male patients may have a firmer and harder ventricular septum. On the contrary, females generally have smaller cardiac dimensions and lower left ventricular mass compared with males, which are associated with shorter native QRS durations and contribute to the shorter post-deployment QRSd. LV enlargement is often accompanied by RV enlargement. A larger ventricular chamber would reduce the stability and support provided by the sheath, leading to potential prolapse during the procedure. Intraventricular conduction delay is more likely to occur in patients with a larger LV, resulting in a persistently wide QRS complex even when selective LBB capture is achieved. Certain methods, such as sheath in sheath, deflectable sheath, or isoproterenol infusion, are helpful to facilitate the LBBAP procedure (33, 34).

### Study limitations

Our study has several limitations. First, this retrospective study was conducted at a single center with a relatively small patient cohort, which may limit the generalizability of our findings. The retrospective design introduces the possibility of selection and performance bias. Patient selection may have been influenced by operator preference, despite standardized procedural protocols. Future multicenter prospective studies with larger sample sizes are warranted to validate these results and enhance their applicability to broader patient populations. Limited by the smaller sample size, we did not classify patients into LBBP, likely LBBP, LVSP, and RVSP for analysis. However, we included all attempts made in these patients with both ideal and inferior results. Comparing the ideal with inferior results provides valuable information for improving LBBAP techniques. Second, all the procedures in our cohort were performed with a Medtronic C315 fixed curve sheath. Outcomes might differ when using stylet-driven leads or sheaths with variable curves, which could provide additional support for challenging anatomies. Further studies using deflectable sheaths, stylet-driven leads, and sheaths with different designs are necessary to validate our findings. Third, we used the criterion of a shorter QRSd to define a more effective LBBAP. While a narrower QRSd intuitively suggests better synchrony in the setting of CSP, it is probably not well tested. For instance, a more apically placed left bundle branch area lead may have a shorter LVAT. Of note, in our study, the LBBAP was performed following the standard protocols which avoided apical septal deployment. Lastly, selective or non-selective capture due to variable pacing outputs is known to affect the QRS duration. Our study only enrolled ECG paced at high output (5 V), which may lead to overestimating QRSd because of non-selective myocardial capture in certain cases, but the pacing thresholds varied in different attempts. Some attempts failed to achieve selective capture. To ensure scientific consistency, we decided on high output pacing which was consistently performed in all procedures.

## Data Availability

The original contributions presented in the study are included in the article/Supplementary Material, further inquiries can be directed to the corresponding author.
